# Microstructure Optimization of Thermoelectric τ_1_-Al_2_Fe_3_Si_3_ via Graded Temperature Heat Treatments

**DOI:** 10.3390/ma17235899

**Published:** 2024-12-02

**Authors:** Ryuta Yurishima, Yoshiki Takagiwa, Ayako Ikeda, Teruyuki Ikeda

**Affiliations:** 1 Graduate School of Science and Engineering, Ibaraki University, 4-12-1 Nakanarusawa, Hitachi 316-8511, Japan; 2 Thermoelectric Materials Group, National Institute for Materials Science, 1-2-1 Sengen, Tsukuba 305-0047, Japan; 3 Research Center for Structural Materials, National Institute for Materials Science, 1-2-1 Sengen, Tsukuba 305-0047, Japan; ikeda.ayako@nims.go.jp

**Keywords:** graded temperature heat treatments, thermoelectric properties, microstructure, phase diagram

## Abstract

To investigate the relationship between microstructure, chemical composition, and thermoelectric properties, we have applied graded temperature heat treatments to recently developed τ_1_-Al_2_Fe_3_Si_3_-based thermoelectric (FAST) materials formed by a peritectic reaction. We investigated microstructures, chemical compositions, and Seebeck coefficients as continuous functions of heat treatment temperature. The τ1 phase can become p- and n-type semiconductors without doping by changing the Al/Si ratio. The Seebeck coefficient was maximized, exceeding |*S*| > 140 μVK^−1^ for both p- and n-type materials, by heat treatment at 1173 K for 24 h through microstructural optimization. These results show that combining the graded temperature heat treatments and spatial mapping measurements of thermoelectric properties gives effective routes to determine the suitable heat treatment temperature for materials with multiphase microstructure.

## 1. Introduction

Physical, mechanical, and thermoelectric properties depend on chemical composition and microstructure [1]. Heat treatments can control microstructure; annealing at high temperatures could remove compositional inhomogeneity due to segregation at the micrometer scale, which may occur in melt-solidified materials. While the heat treatment conditions could be estimated by calculation if the diffusion coefficient is known, diffusion coefficients are unknown in many cases. Therefore, based on a known phase diagram, if available, a trial-and-error approach is often employed in various heat treatments such as homogenizing chemical compositions, promoting phase transition, or controlling microstructures. Exploring optimal heat treatment conditions is, in general, time-consuming and labor-intensive. In order to efficiently optimize heat conditions, taking a look at high throughput techniques, there are some, but limited, studies found using graded temperature heat treatments [2,3]. Ning et al. developed a graded temperature heat treatment to prepare a Ni base superalloy (FGH4096) with structural gradient [2]. Wei and Zhao used a similar heat treatment to study the solid state precipitation in Fe-Cr-Mo steel over a wide range of temperatures [3].

Recently, a new thermoelectric material, “FAST materials” (Fe-Al-Si Thermoelectric materials), has been developed [4,5]. It is a ubiquitous material based on the τ_1_–Al_2_Fe_3_Si_3_ single phase [6]. The τ_1_ phase has such a wide compositional range that allows variation in the Al and Si concentrations, *x*_Al_ = 21.0–41.5 at.% and *x*_Si_ = 41.5–21.0 at.%, with a limited range of the Fe concentration (*x*_Fe_: 37.5–38.5 at.%) [6]. One can control the carrier conduction behavior of FAST materials from p- to n-type depending on the Al/Si ratio [7,8], and the maximum power factor S^2^σ is ~880 µWm^−1^K^−2^ [9]. For its abundance, environmentally friendly characteristics, and excellent thermoelectric properties, FAST is a promising material that would be utilized as autonomous power supplies for IoT devices in an advanced information society [10]. In order to realize such widespread use of this material, optimization of material properties is desired from various points of view, including microstructure control; the heat treatment condition for FAST materials has not been refined.

The τ_1_ phase is formed by a peritectic reaction between the FeSi and liquid phases [6], and hence an ordinary solidification processing results in the peritectic microstructure. Therefore, to prepare material with τ_1_ single phase, it is necessary to carry out heat treatments. To optimize heat treatment conditions, we conducted graded temperature heat treatments to evaluate thermoelectric performance and microstructure as continuous functions of heat treatment temperature.

## 2. Materials and Methods

### 2.1. Sample Preparation

Ingots of FAST materials with the nominal compositions of Fe_38_Al_22+*x*_Si_40-*x*_ (*x* = 0 and *x* = 2.2 for n- and p-type, respectively) were synthesized using an induction heating furnace (NEV- SM04N; Nissin Giken Co., Iruma, Japan) under an argon atmosphere. After crushing the ingots, gas atomization produced a powder sample. Powder with a particle size of less than 45 μm was sintered in a graphite die with a diameter of 15 mm by spark plasma sintering method (LABOX-110MC; SinterLand, Inc., Nagaoka, Japan) at 1193 K under a uniaxial pressure of 57 MPa and an argon atmosphere. After grinding the sample surface to remove surface impurities, the sample was cut into a rod shape with a 6 mm diameter and a 10 mm height using an electric discharge machine. Microstructures after sintering were observed by a scanning electron microscope (SEM) equipped with an electron-probe microanalyzer with wavelength dispersive X-ray spectroscopy (EPMA; EPMA-8050G; Shimadzu Corp., Kyoto, Japan).

### 2.2. Heat Treatments Under Temperature Gradation

**Figure 1:**
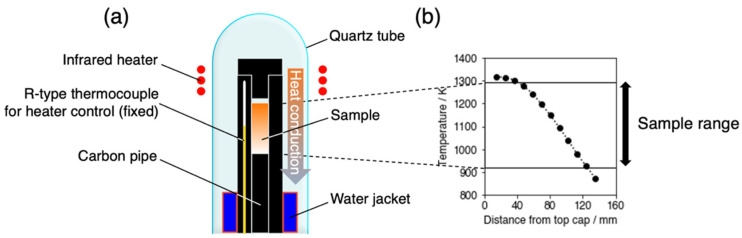
Schematic view of the furnace used for graded temperature heat treatments (**a**) and its temperature distribution (**b**).

Samples thus prepared were subject to heat treatments in a temperature gradation. A schematic view of the graded temperature furnace is shown in Figure 1a, and the temperature distribution in the furnace measured with an R-type thermocouple is shown in Figure 1b. Samples were heat treated in a graphite container under Ar gas for 24 h. The temperature gradation was formed between the heated part by an infrared lamp and the cooled part by water-cooling. Eight samples were stacked vertically in the graphite container and set in the graded temperature region from ~873 K to 1323 K. Heat treatment temperature was estimated using the distance from the cap of the furnace compared with the temperature distribution shown in Figure 1b. The preciseness in measuring the distance is estimated to be ±1 mm, which correspond to the preciseness of temperature of ±5 K based on the temperature distribution. The accuracy of the temperatures thus measured was checked using the solvus in the Ni-Al system; a rod sample with the Ni-14at.%Al composition was subject to a graded temperature heat treatment, and the solvus temperature determined from the position of the boundary between the regions where precipitates are observed and not observed was 1097 K, which is in good agreement with that in the reported Al-Ni phase diagram [11], 1098 K.

After the heat treatment, the samples were water-cooled to freeze stable phases under heat treatment temperatures by dropping them into water.

### 2.3. Characterization

The samples were cut in the longitudinal direction, and the cut surfaces were polished using abrasives of #320, #400, #600, followed by diamond slurries with particle sizes of 9 μm, 3 μm, and 0.05 μm, respectively. The microstructure was then observed, and the chemical compositions were determined using SEM and EPMA as functions of annealing temperature. Phase fractions were determined from the microstructural images. The Seebeck coefficient was measured near room temperature as a function of the distance from the bottom edge. The sample was mounted on a temperature-controlled copper plate to provide uniform heat throughout the sample. The temperature difference was created between a thermocouple with a small thermal mass located at a corner of the sample and a cold scan probe with a large thermal mass, which locally draws heat from the sample surface to create the local temperature difference Δ*T*. The procedure is similar to that described in [12,13]. In this work, the spatial resolution of the Seebeck coefficient measurements is ~150 µm, reflecting the wire diameter of the T-type thermocouple used in this work. At each distance from the top cap of the furnace, measurements were done for five different points and the five values were averaged.

## 3. Results and Discussion

**Figure 2:**
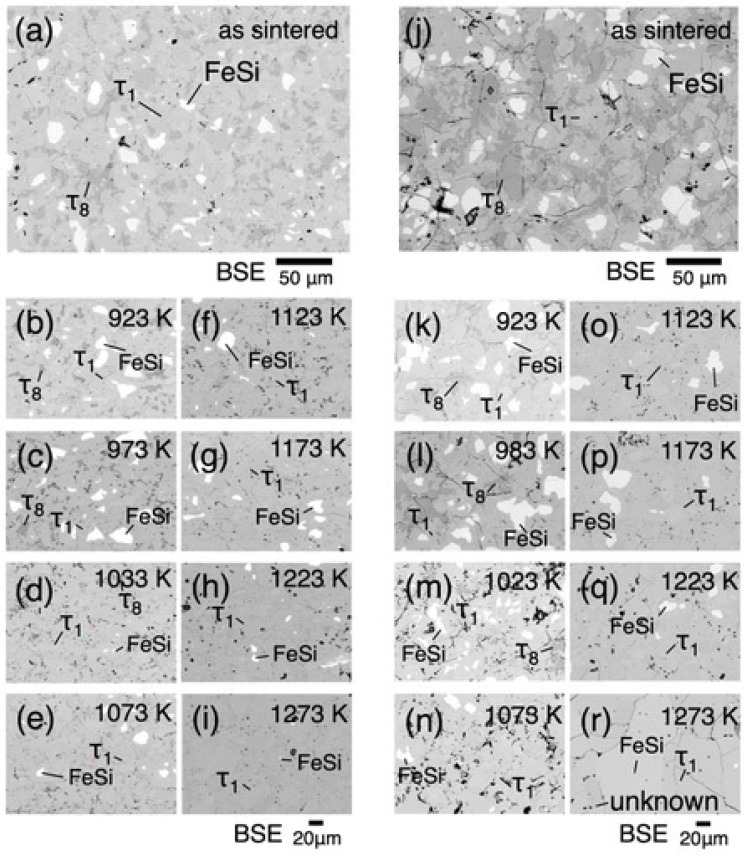
Microstructures of as-sintered p-FAST (**a**) and n-FAST (**j**). Figures (**b**–**i**) and (**k**–**r**) show microstructures taken from the p-FAST and n-FAST, respectively, after graded temperature heat treatments. Corresponding temperatures of the heat treatments are shown in the respective images.

Compositions of the sintered samples measured by EPMA are Fe_38.7±0.7_Al_23.6±0.5_Si_37.7±0.4_ for p-FAST and Fe_39.9±0.7_Al_21.8±2.2_Si_38.4±1.8_ for n-FAST, where the error ranges correspond to the standard deviations of seven measured points. Three-phase microstructures with tens of micrometers size scales are observed in the as-sintered samples, as shown in Figure 2a,j. Since τ_8_ and FeSi phases cannot be in equilibrium with each other according to the equilibrium phase diagram [14], the three-phase state is thought to be in a non-equilibrium state reflecting the solidification microstructure after the solidification processing by gas atomization.

**Figure 3:**
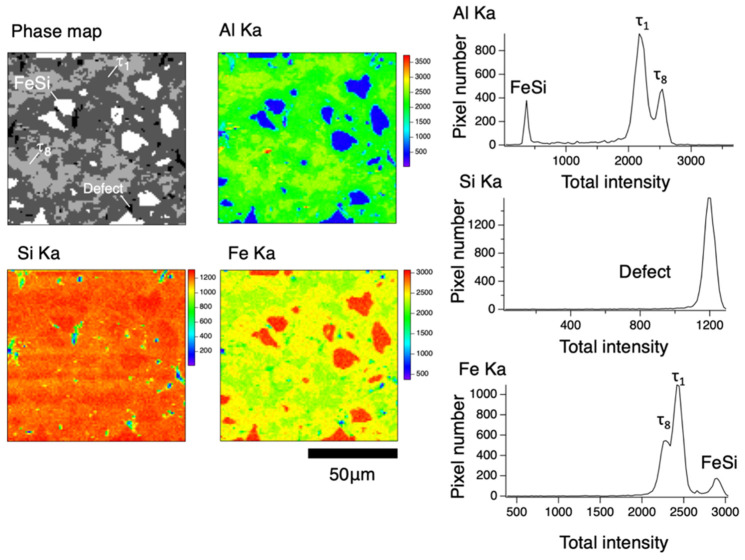
The analysis to evaluate the volume fractions of constituent phases for the region heat-treated at 993 K in the n-FAST, as an example, after the graded temperature heat treatments.

**Figure 4:**
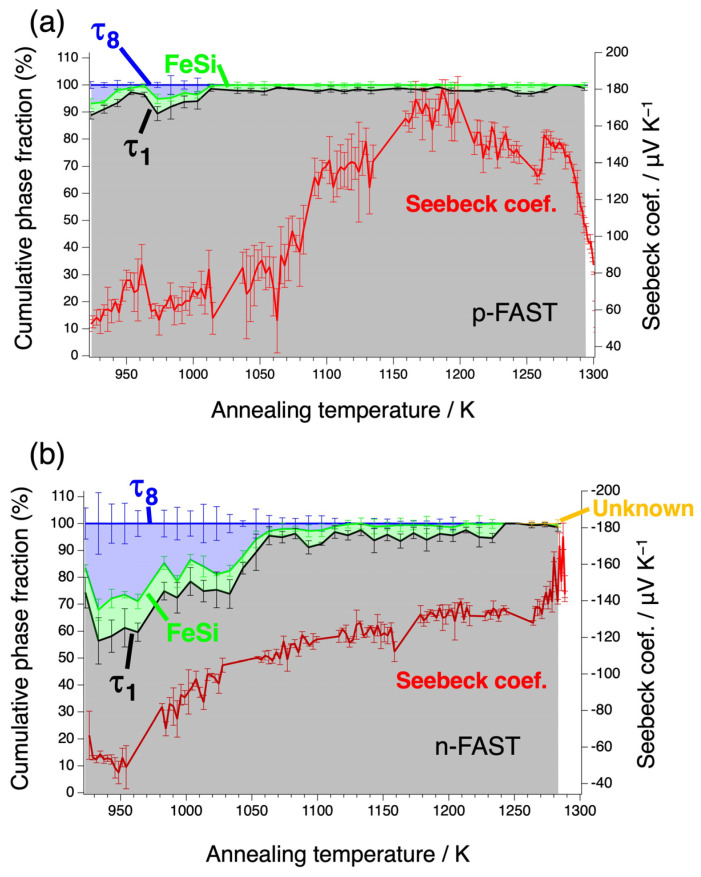
Seebeck coefficients mapped at room temperature and cumulative phase fractions after the graded temperature heat treatment as functions of temperature for p- FAST (**a**) and n- FAST (**b**).

**Figure 5:**
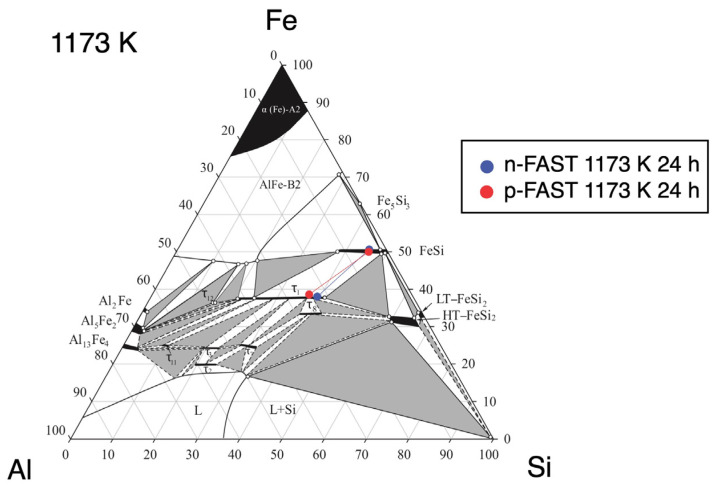
Compositions measured in the p- and n-FAST samples after the graded temperature heat treatment plotted with the reported Al-Fe-Si phase diagram [6].

Microstructures after the heat treatment are shown for various heat treatment temperatures in Figure 2b–i,k–r. The chemical compositions of phases observed in the microstructures at respective temperatures are shown in Figure S1 together with the phase diagram [6]. Three-phase microstructures consisting of τ_1_, τ_8_, and FeSi are observed at temperatures up to 1033 K in both the p- and n-type samples, while the volume fraction of the τ8 phase decreases with increasing temperature. Above 1033 K, microstructures are composed of the τ_1_ and FeSi phases with a decreasing trend in the FeSi phase fraction. In addition, there are precipitates at grain boundaries at 1273 K in the n-FAST sample, while this phase has not been identified. The phase fractions were evaluated using X-ray intensity maps for each element obtained by EPMA; results of analysis from the n-type sample at 993 K are shown as examples in Figure 3. Since the samples were prepared by sintering, significant fractions of voids were observed. The phase fractions were evaluated within solid regions. The cumulative phase fractions and Seebeck coefficients are shown in Figure 4. Absolute values of the Seebeck coefficients of both p- and n-types increase up to around 1173 K. As the spatial resolution of the Seebeck coefficient measurements is ~150 µm, which is larger than the length scale of the phase variation shown in Figure 2, each Seebeck coefficient at a single measured point represents the average values of multiple phases shown in Figure 2. At each distance from the top cap of the furnace, measurements were done for five different points and the five values were averaged. The standard deviations of five measured values were used to show the range of error in Figure 4. The trend of absolute values of the Seebeck coefficients is accompanied by the increase in the τ_1_ fraction and the decrease in the τ_8_ fraction, and is reasonable because the τ_8_ phase shows metallic behavior and hence is expected to exhibit a low Seebeck coefficient while the τ_1_ is known for its excellent thermoelectric properties arising from the formation of a narrow gap near the Fermi level according to first principle calculations [8]. Such variations of the phase fractions can be attributed to atomic diffusion; diffusion distance due to the 24 h annealing is not large enough at low temperatures up to 1173 K compared to the size scales showing inhomogeneity in the as-sintered state shown in Figure 2, while it is large enough to homogenize the samples and achieve the equilibrium compositions of phases at 1173 K and above. Actually, the compositions measured from respective phases are consistent with the reported phase diagram, as shown in Figure 5 for 1173 K [14].

**Figure 6:**
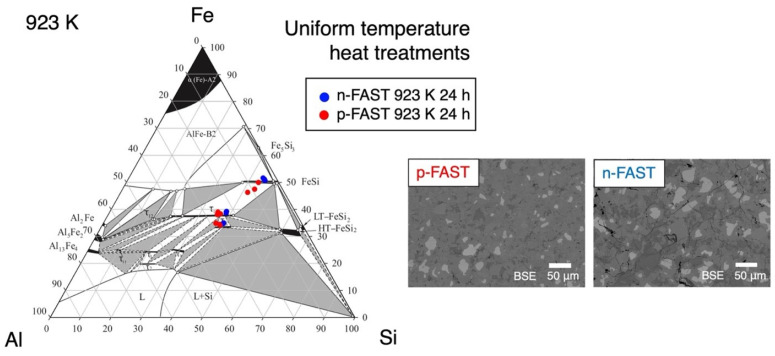
Microstructures and compositions obtained in the sample heat-treated using a uniform temperature furnace at 923 K for 24 h.

To examine the validity of the above arguments, we first check the validity of the temperatures within the samples during the graded temperature heat treatments. Samples at the same compositions Fe_38_Al_22+*x*_Si_40-*x*_ (*x* = 0 and *x* = 2.2 for n- and p-type, respectively) were annealed in a uniform-temperature furnace. The microstructure and the chemical composition of the sample annealed at 923 K for 24 h are shown in Figure 6, where microstructures with the three phases, τ_1_, FeSi, and τ_8_, are observed. This phase composition is consistent with that in the samples heat-treated in the graded temperature. This result shows that the temperature during the graded temperature heat treatment is not contradictory to that during the uniform temperature annealing.

Next, we consider whether the phase composition, τ_1_, FeSi, and τ_8_, at 1023 K and lower temperatures in Figure 2 are really of nonequilibrium states. According to Du et al. [15], both experiments and CALPHAD calculations show that, on the Si-rich side of the τ_1_ phase, τ_1_ phase is in equilibrium with the FeSi and FeSi_2_ phases, or with τ_8_ and FeSi_2_ phases at 1000 K, and hence the FeSi, τ_1_, and τ_8_ phases are not in phase equilibrium. Therefore, the three-phase state with the τ_1_, FeSi, and τ_8_ phases at temperature lower than 1000 K should be in a non-equilibrium state. This means that the reason why the microstructure of the three phases of τ_1_, FeSi, and τ_8_ is observed at 1023 K and lower temperatures is that the annealing of this material at temperatures lower than 1000 K for 24 h does not give a long diffusion distance enough to equilibrate the samples remaining in the non-equilibrium state due to solidification processing, including the peritectic reaction.

A significant increase in *S* for n-FAST and a decrease in *S* for p-FAST is recognized with the absolute values of the Seebeck coefficient above ~1273 K. This region may be affected by compositional change due to annealing at high temperatures.

To consider these variations in Seebeck coefficient above 1273 K, first we focus on the effect of grain boundary phase observed in n-FAST as shown Figure 2r. According to a previous study [8], electronic states of phases other than τ_1_ and τ_12_ in the Fe-Al-Si phase diagram are metallic, which inherently results in lower Seebeck coefficients than that of τ_1_. Therefore, the increase in the absolute value of the Seebeck coefficient of n-FAST above 1273 K cannot be explained as the contribution of such metallic phases. On the other hand, the decrease in the absolute value of the Seebeck coefficient above 1273 K in p-FAST (Figure 4a) is unlikely to be attributed to grain boundary phases, even if their Seebeck coefficients are assumed to be very small because the area fraction of the grain boundary phase is too small to explain the observed decrease in Seebeck coefficient.

Assuming that the grain boundary phase is the τ_12_ phase and Seebeck coefficient of the τ_12_ phase is significantly higher than that of the τ_1_ phase, it may contribute to the increase of the Seebeck coefficient above 1273 K in n-FAST (Figure 4b). According to the phase diagram [6], the composition of the τ_12_ phase is Al-richer and slightly Fe-deficient compared to τ_1_, being consistent with the darker contrast observed in SEM. If the grain boundary precipitates in n-FAST (Al-deficient) are really the τ_12_ phase, it should be more likely that the same thing happens in p-FAST (Al-rich). However, no grain boundary precipitates are observed in p-FAST.

Thus, the drastic variations of the Seebeck coefficients above 1273 K are neither due to the contributions from the metallic phases nor the τ_12_ phase in the Al-Fe-Si system.

Considering this, the primary cause of the variation of the Seebeck coefficient observed above 1273 K in Figure 4a,b is likely to be an increase in the Al/Si ratio within the τ1 phase itself, rather than the influence of grain boundary phase.

According to the Seebeck coefficient estimated by a first principle calculation as a function of chemical potential [4], such behavior of the Seebeck coefficient, that is, increase for n-FAST and decrease for p-FAST in the absolute values by annealing, can occur with the shift to lower chemical potentials for both n- and p-types, which corresponds to the shift of the chemical compositions in the Al-rich direction and cannot be explained by Al evaporation. Therefore, the results of compositional analysis of samples heat-treated under a temperature gradient indicate that at 900 °C diffusion occurs, but the effect of temperature gradient diffusion does not occur, while at higher temperatures, the effect of temperature gradient diffusion may occur. In addition, there is not enough diffusion at lower temperatures because microstructures at lower temperature should be non-equilibrium. The reason for such a compositional variation might be diffusion due to temperature gradient, i.e., thermal diffusion [16]. However, the argument cannot be conclusive at present and is beyond the scope of this work. Thus, in general, if the temperature of heat treatment is too high, unexpected effects may be caused, and hence one needs to choose the right temperature for heat treatments. These results show that combining the graded temperature heat treatments and spatial mapping measurements of thermoelectric properties gives effective routes to determine the suitable heat treatment temperature for materials with multiphase microstructure.

## 4. Conclusions

We have applied graded temperature heat treatments to p- and n-FAST materials prepared by induction melting followed by gas atomization and sintering, which enabled us to examine microstructures, chemical compositions, and Seebeck coefficients as continuous functions of temperature. As a result, the Seebeck coefficient was improved by heat treatment at 1173 K for 24 h. This is accompanied by the microstructure change from a non-equilibrium state containing the τ_1_, FeSi, and τ_8_ phases to an equilibrium state containing the τ_1_ and FeSi phases. Thus, eliminating the τ_8_ phase, which is harmful to thermoelectric properties, effectively optimizes thermoelectric properties. Heat treatments under a temperature gradation give effective routes to determine the proper temperature of heat treatments.

## Data Availability

The raw data supporting the conclusions of this article will be made available by the authors on request.

## Supplementary Materials

The following supporting information can be downloaded at: https://www.mdpi.com/article/10.3390/ma17235899/s1, Figure S1: Compositions of phases observed in n-FAST after the graded-temperature heat treatments.
