# Supplementary material for: Microstructure Optimization of Thermoelectric τ1-Al2Fe3Si3 via Graded Temperature Heat Treatments
Source: Materials (Basel). 2024 Dec 2;17(23):5899. doi: 10.3390/ma17235899 (PMC11643797; doi:10.3390/ma17235899)
Supplement: Supplementary file 1 [file materials-17-05899-s001.zip › materials-3325785-supplementary.pdf]

Supplementary materials

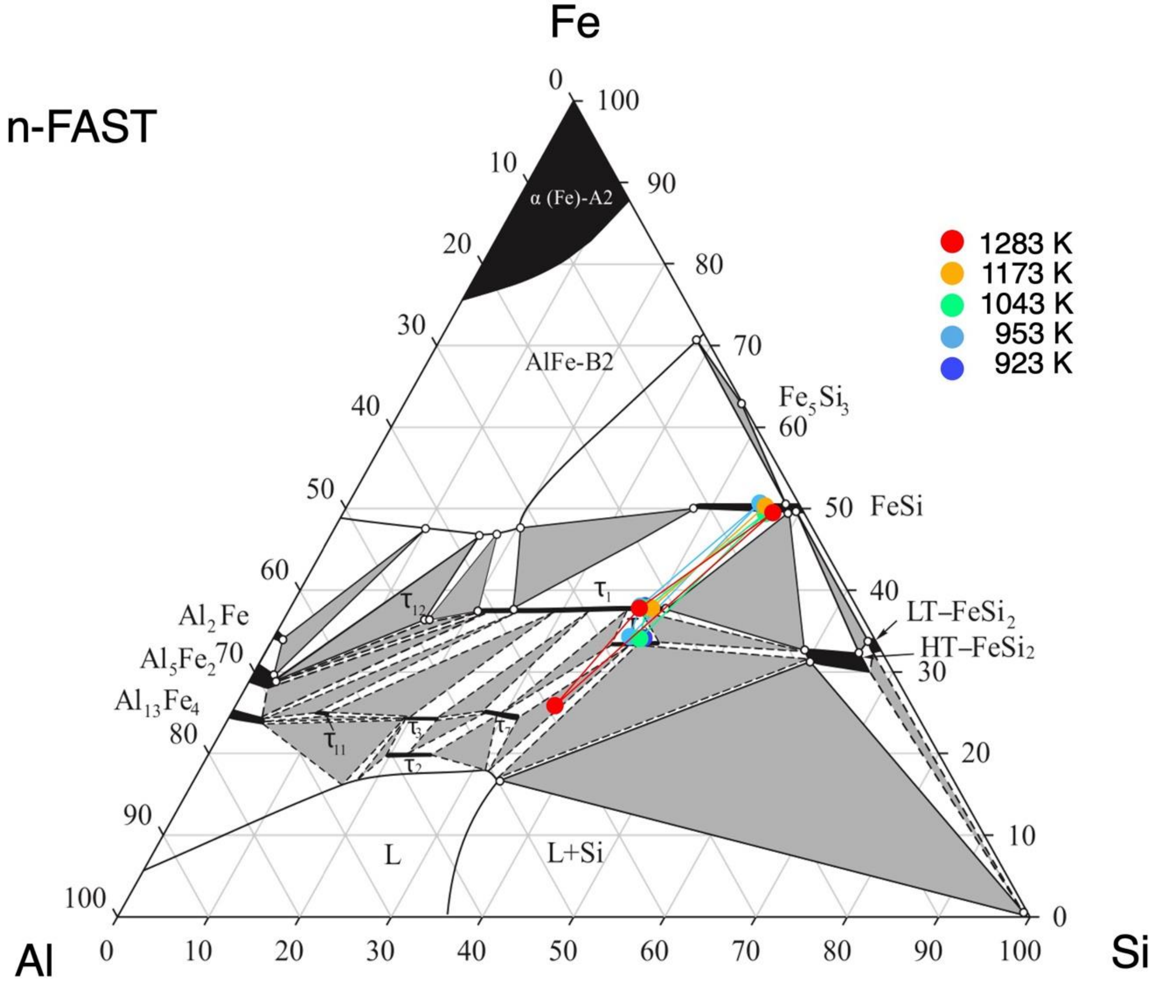

Figure S1. Compositions of phases observed in n-FAST after the graded-temperature heat treatments.
